# 3D engineered scaffold for large-scale Vigil immunotherapy production

**DOI:** 10.1038/s41598-024-65993-3

**Published:** 2024-07-05

**Authors:** Fabienne Kerneis, Ernest Bognar, Laura Stanbery, Seongjun Moon, Do Hoon Kim, Yuxuan Deng, Elliot Hughes, Tae-Hwa Chun, Darron Tharp, Heidi Zupanc, Chris Jay, Adam Walter, John Nemunaitis, Joerg Lahann

**Affiliations:** 1 Gradalis, Inc, Dallas, TX 75006 USA; 2 University of Michigan Biointerfaces Institute, Ann Arbor, MI 48109 USA; 3 Department of Metabolism, Endocrinology and Diabetes, University of Michigan Medical School, Ann Arbor, MI 48109 USA; 4 Department of Gynecologic Oncology, Promedica, Toledo, OH 43560 USA

**Keywords:** Cell expansion, Vigil, Immunotherapy, Autologous tumor cell therapy, Ovarian cancer, Ovarian cancer, Cell growth

## Abstract

Previously, we reported successful cellular expansion of a murine colorectal carcinoma cell line (CT-26) using a three-dimensional (3D) engineered extracellular matrix (EECM) fibrillar scaffold structure. CCL-247 were grown over a limited time period of 8 days on 3D EECM or tissue culture polystyrene (TCPS). Cells were then assayed for growth, electroporation efficiency and Vigil manufacturing release criteria. Using EECM scaffolds, we report an expansion of CCL-247 (HCT116), a colorectal carcinoma cell line, from a starting concentration of 2.45 × 10^5^ cells to 1.9 × 10^6^ cells per scaffold. Following expansion, 3D EECM-derived cells were assessed based on clinical release criteria of the Vigil manufacturing process utilized for Phase IIb trial operation with the FDA. 3D EECM-derived cells passed all Vigil manufacturing release criteria including cytokine expression. Here, we demonstrate successful Vigil product manufacture achieving the specifications necessary for the clinical trial product release of Vigil treatment. Our results confirm that 3D EECM can be utilized for the expansion of human cancer cell CCL-247, justifying further clinical development involving human tissue sample manufacturing including core needle biopsy and minimal ascites samples.

## Introduction

Vigil is a fully personalized, patient-specific cancer immunotherapy comprised of autologous tumor cells that are transfected via electroporation with the Vigil plasmid. The Vigil plasmid delivers GM-CSF and a bifunctional short hairpin RNA designed to knock down furin (bi-shRNA^*furin*^) expression^1,2^. Furin is the critical enzyme responsible for downstream processing of TGF-β. GM-CSF serves as an immune stimulatory cytokine that enhances anti-tumor response through upregulation of antigen presentation and T cell activation. By utilizing the patient’s tumor cells, this triple-function immunotherapy serves to expose the full repertoire of patient-specific tumor clonal neoantigens to the immune system, increase immune activity at the intradermal injection site in response to clonal neoantigen dendritic cell response with the aid of increased GM-CSF expression. Additionally knockdown of furin inhibits activity of key TGF mediators of tumor cell immune evasion, TGF-β1 and TGF-β2.

The clinical safety of Vigil is well documented, with no ≥ Grade 3 adverse events reported in clinical trials (1433 doses administered to 230 patients)^3–9^. A Phase I, open-label trial established the safety and efficacy of Vigil involving advanced solid tumors and demonstrated an overall survival (OS) benefit compared to historical expected survival^6,7^. Patient survival advantage was correlated with Vigil-induced immune activation, assessed by the γIFN-ELISpot assay^7^. These results were corroborated by a Phase II clinical trial investigating Vigil efficacy as a frontline maintenance therapy in newly diagnosed Stage IIIb/IV ovarian cancer patients who achieved complete clinical response following induction therapy with carboplatin/paclitaxel. Vigil demonstrated improved recurrence-free survival (RFS) of 7.4 months (HR 0.43; p = 0.0165) between Vigil (n = 31) when used as maintenance therapy compared to no treatment standard of care^3^. Follow-up performed 3 years later revealed a continued significant durable survival advantage with Vigil. Clinical benefit was also correlated with positive ELISpot response after Vigil^4^. Moreover, NanoString assessment further demonstrated OS advantage to Vigil in association with high tumor inflammation score (TIS ≥ 6) in comparison to low TIS^10^.

On the basis of these results, a Phase IIb, double blind, placebo-controlled trial of Vigil vs. placebo was performed in newly diagnosed Stage IIIb/IV resectable ovarian cancer patients after achieving complete response following surgery and chemotherapy. In this trial, 91 patients received either Vigil (1 × 10^7^ cells per dose) or placebo for a minimum of four and up to twelve doses administered once a month^9^. A subset of patients with the homologous recombination proficient (HRP) profile demonstrating proficient DNA repair capacity which preserves clonal neoantigen expression, achieved RFS (HR 0.386, p = 0.007) and OS (HR 0.342, p = 0.019) advantage with Vigil^5^. Two year OS in the HRP Vigil treated group was 92% vs. 55% (p = 0.002) in the control group and subsequent long term follow up at 3 years revealed durable OS advantage persistence in this group of 70% vs. 40% (p = 0.019) to Vigil^8^. ENTPD1-high and p53mu tumor biomarkers were also separately shown to be predictive of OS and RFS benefit with Vigil treatment vs. placebo in an initial exploratory analysis^11,12^. HRP patients with high ENTPD1 demonstrated RFS of 21.1 months for Vigil and 5.6 months for placebo (p = 0.004)^12^. Further clinical investigations of Vigil are targeted at HRP cancers, especially HRP ovarian cancer, where the current standard of care treatments bring no survival advantage.

Vigil manufacture is simple and robust for indications like ovarian cancer where tumor tissue is obtained through standard of care surgery. However, one barrier to the successful production of Vigil is that surgically-excised tumor tissue, particularly in other indications, does not always contain enough tumor cells for successful Vigil construction. Currently, successful Vigil manufacture requires 10–30 g of tumor tissue (cumulative). Additionally, poor tissue quality, leading to poor cell recovery, can also result in insufficient cells for successful Vigil manufacture. Therefore, an attractive direction to broaden the use of Vigil would be the integration of limited tumor cell expansion which would allow for the use of smaller tumor samples, such as those removed via needle core biopsy. Needle core biopsy does not require general anesthesia which is currently necessary for surgical harvest of disease undergoing Vigil manufacturing. Conventional cell expansion methods using 2D culture are limited by changes in phenotypic and genotypic signatures. While other 3D methods including microfluidic devices and spheroids have been developed, these are typically utilized on a smaller scale^13–15^. Therefore, 3D EECM scaffold technology represents a scalable scaffold platform that limits phenotypic and genotypic drift to provide robust cell number expansion for Vigil manufacture^16^.

Feasibility of the 3D EECM for propagating cells has been demonstrated using mouse cell line, CT-26 as well as CCL-247 human colorectal cell line^16^. Encouragingly, we observed rapid cell expansion on the 3D EECM platform with expansion levels superseding those of conventional 2D cell culture methods because of the large surface area and ultra-high porosity of the 3D scaffolds used to support the EECM^17^. We also demonstrated successful product release achievement similar to what has been demonstrated with Vigil in FDA approved randomized control trial^5,8,9^. This initial work supports 3D EECM as a new tool for cell expansion, with exciting potential in the context of autologous cell therapies, where treatment can often be limited by insufficient cell numbers.

## Methods

### 3D scaffold preparation and Fn deposition

The preparation of the 3D EECM followed methodologies detailed in our previous research^16^. The 3D EECM consists of two essential elements: a 3D hyper-porous polycaprolactone (PCL, average Mn 45,000 purchased from Sigma Aldrich, USA) scaffold and an a fibrillar fibronectin matrix (Fn, from human plasma, purchased from Sigma Aldrich, USA).

The 3D hyper-porous PCL scaffold was fabricated by melt electrospinning using a Regen Hu 3D discovery bioprinter (R-GEN-100, Regen hu, USA). Using this approach, we created scaffold structures based on 200 μm thick PCL fibers organized into 3D stacks of various geometries^18,19^. Using these tessellated scaffolds, a fibrillar Fn network was deposited using shear-induced fibrillogenesis^16,20,21^. Fn deposition via shear-induced fibrillogenesis occurred in a custom-made chamber system. To secure larger coverage of the fibrillar fibronectin on the PCL scaffold, the shear-induced fibrillogenesis was repeated twice for each PCL scaffold.

### Cell culture and seeding onto 3D EECM and TCPS

Cell culture of colon cancer cell line CCL-247 (purchased from ATCC, Gaithersburg, MD, USA) were maintained in McCoy’s 5A (ATCC) medium supplemented with 10% heat inactivated FBS and 2 mM l-glutamine.

For 2D cell culturing, CCL-247 cells were seeded on T225 tissue culture flask supplied with appropriate culture media and were split/expanded based on confluency. Cells were detached using 0.2% trypsin–EDTA solution when cells reached confluency (80–90%). For 3D scaffold culturing, scaffolds were first placed in 12-well plate, one scaffold per well with identical culture media used for 2D. 2.5 × 10^5^ CCL-247 cells (5% of confluency) were seeded onto the scaffold. The media was changed every 2 days.

CCL-247 cells at passage 5 from the same tissue culturing flask were split into two parts as the seeding cells for 3D and 2D culturing.

### Cell dissociation

After cell density reached the desired confluency, CCL-247 cells were harvested after trypsin–EDTA treatment in 2D culturing flask, or collagenase-I treatment in a 3D scaffold culturing matrix.

### Cell proliferation determination

To assess cell proliferation, we employed the Resazurin-based in vitro toxicity assay (TOX8, purchased from Abcam, USA). Solutions for the TOX8 assay were prepared by mixing 10% (v/v) TOX8 reagent with 90% (v/v) of the harvesting media.

Following the aspiration of media from the well, 2 mL of the prepared TOX8 solution was added. The wells were then incubated at 30 °C for 1 h. After incubation, the color-changed TOX8 solutions were transferred into a 96 deep well plate and centrifuged to remove any residual cells in the solution.

The clear supernatant was then carefully transferred to a black, clear-bottom 96-well plate, ensuring that no air bubbles were introduced. The samples fluorescence was then measured, utilizing an excitation wavelength of 560 nm, and observing the increase in fluorescence at an emission wavelength of 590 nm.

### Electroporation test using GFP plasmid

The ability of transfection of the cells from 3D EECM was confirmed using the electroporation of GFP plasmid into the cell. The secured cells were dispersed into a PBS solution with 1.0 × 10^7^ cells/mL of concentration. The GFP plasmid was added at 1 mg/mL of concentration. The electroporation was conducted with 300 V of voltage, and 1000 μF of capacitance in a 0.4 cm electrode-gaped Gene pulser cuvette (purchased from Bio-Rad, USA) using GenePulser Xcell (Bio-Rad, USA). Following the electroporation, the cells were plated onto the T25 flask and incubated for 1 day. To confirm the transfection of the GFP plasmid, we conducted flow cytometry (Bio-Rad ZE5, USA).

### Vigil manufacturing process

Vigil immunotherapy manufacturing was performed following standard manufacturing protocol. Cells were harvested and resuspended in X-VIVO 10 medium with gentamicin (40 µg/mL) added for cell counting post chemical dissociation. Prior to transfection, 1.33 × 10^6^ cells were taken as a control for the QC cytokine release assay (triplicate). QC cells were cultured in 1.0 mL X-VIVO 10 medium. The remaining processed cells were centrifuged and resuspended at a concentration of 4.0 × 10^7^ cell/mL and transferred into a 4 mm gap electroporation cuvette and mixed with 50 µg Vigil plasmid DNA (Waisman Biomanufacturing, Madison, WI, USA). Cells were electroporated at 300 V, 1000 µF with indefinite resistance exponential decay protocol. After electroporation, cells were transferred to a T225 flask with X-VIVO 10 medium and cultured for 18 h ± 4 h at a concentration of 1 × 10^6^ cells/mL in an incubator at 37 °C, 5% CO_2_.

Post overnight culture, cells were harvested and counted. A minimum of 70% viability is required to proceed with irradiation step (100 Gy irradiation dose using Rad Souse RS3400, Atlanta, GA, USA). See Table 1. Post irradiation step, 1.33 × 10^6^ cells were taken and cultured in 1 mL X-VIVO 10 medium for the QC release cytokine assay (triplicate). For all QC cultures, supernatant was collected after 4 days of culture. Post irradiation, the in-process cells were aliquoted into cryovials and frozen with 10% DMSO at a concentration of 1.0 × 10^7^ cells in 1 mL. Following a controlled-rate freezing process, cryovials were transferred the cells to liquid nitrogen storage tank for long term storage.

**Table 1:** Comparison 2D and 3D manufacturing results.

Source	Dissociation reagent and concentration	Day 1 harvested cells number	Day 2 post overnight culture recovery (%)	Day 2 viability ≥ 70% intact cells	Number of product vial created ≥ 4 product vials
Standard 2D	Trypsin 0.25%	126 × 10^6^	57	94	6
3D EECM	Collagenase 0.5%	120 × 10^6^	55	91	5

### Vigil plasmid verification assay

#### GM-CSF and TGF-β1 level measurement

Supernatant samples from the Vigil manufacturing process were tested using the ELLA platform (automated ELISA system from ProteinSimple (Wallingford, CT) to detect GM-CSF and TGF-β1 levels in cell culture supernatant samples. The ELLA result (pg/mL) was normalized against the cell culture density (1.33 × 10^6^ cells/mL) to generate the final potency result (pg cytokine detected/10^6^ cells in culture).

The human GM-CSF detection kit [SPCKB-PS-000493 from ProteinSimple (Wallingford, CT)] used for GM-CSF cytokine level measurement. Briefly, samples were diluted 1:1000 fold with X-VIVO medium and mixed with diluent SD13 at 1:1 ratio. Human GM-CSF standards (ProteinSimple, Santa Clara, CA) were also tested as positive controls. The GM-CSF concentration in the sample was calculated by GM-CSF standard curve generated from the same cartridge.

For TGF-β1 measurement, the human TGF-β1 detection kit (ProteinSimple, Wallingford, CT) was used. Briefly, the human TGF-β1 active form was first released by addition of 1/2 volume 1.0 N HCl into the X-VIVO medium sample. After denaturing for 10 min at room temperature, the medium sample was neutralized by addition of 1.2 N NaOH at an equal volume of HCl was added. After activation, medium samples were mixed with RD5P diluent from kit at 1:1 ratio. 50 µL of the mixture was loaded into human TGF-β1 detection cartridge with human TGF-β1 standards (ProteinSimple, Wallingford, CT, USA). The TGF-β1 concentration in the sample was calculated by TGF-β1 standard curve generated from the same cartridge. The both GM-CSF and TGF-β1 standards and results were first analyzed to meet QC release criteria before further analysis.

#### Determination of intact cells and cell viability

Viable cells were detected by a trained operator manually using a validated proprietary method under regular microscopy in phase contrast setting with Trypan Blue stain. Cells that are considered “live” or “viable” must show membrane integrity; a cell with visual disruption of the membrane would be considered a non-viable or dead cell. Cell counts were performed prior to transfection at Day 1 and before irradiation at Day 2 for final vialing. Viability is assessed at two time points including the Day 1 of manufacturing and Day 2 but has to pass the 70% criteria only at Day 2.

#### Determination of purity/safety (endotoxin, sterility, mycoplasma)

2D and 3D samples were sterility tested at Gradalis, using the BacT Alert automated microbial detection system. Vialed samples were inoculated into aerobic and anaerobic bottles and incubated for 7 days at 37 °C.

Other QC samples were submitted to ATCC (Manassas, VA, USA) for short tandem repeat (STR) profile analysis. 2D and 3D samples were tested for endotoxin by kinetic chromogenic assay [Charles River (Houston, TX, USA)] and mycoplasma testing by 28-day direct culture combined with DNA fluorochrome staining assay [Bionique (Saranac Lake, NY)].

#### Identity testing

Samples were sent to Frontage Labs (Exton, PA) for identity testing with custom designed PCR assay designed to specifically detect Vigil plasmid in the final product.

A custom PCR assay was designed by Frontage Labs to specifically detect Vigil plasmid present in the final product. Total DNA is extracted from Vigil final product and checked for quantity (260/280) and quality (gel analysis). PCR primers were designed and verified to specifically react against Vigil plasmid and not cross react with other Gradalis vectors, nor genomic DNA. The PCR assay has a quantitative range of 1000–100,000,000 Vigil copies per reaction. The PCR assay includes primers against the house-keeping gene ACTA1 to confirm the presence of genomic DNA in the sample. The Vigil copies detected are normalized per ACTA1 copies to calculate Vigil copies per cell.

## Results

### Successful expansion of CCL-247 cells using 3D EECM

**Figure 1:**
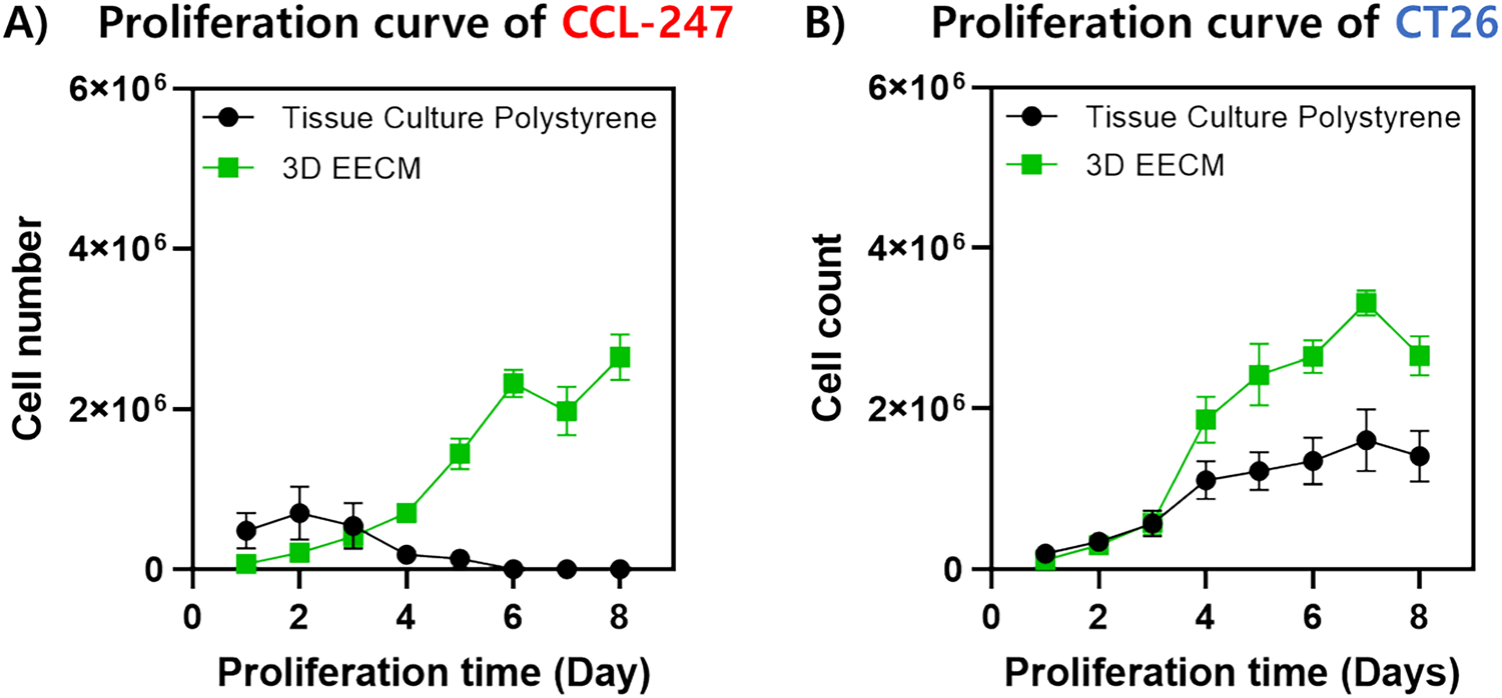
Proliferation curves for CCL-247 (**A**) and CT-26 (**B**) cells. Cells were grown on 3D EECM or TCPS for 8 days. The cell proliferation curve was quantified using the TOX8 assay daily.

Previous work demonstrated the ability of the 3D EECM to support robust expansion of murine colorectal cell line CT-26 which is a commonly used cell line for immune-related studies^16,22^. Next, we wanted to investigate the ability of 3D EECM to support the growth of the human colorectal carcinoma cell line CCL-247. Tissue culture polystyrene (TCPS), which is used in traditional 2D cell culture was used as a control. To track the proliferation of the cells, we used a resazurin-based in vitro toxicity assay (TOX8 assay)^23^. CT-26 cells were also used as a control. We observed cell rapid proliferation that was consistent with previously published work (Fig. 1B). Our study reveals that our intricately designed fibrillar 3D EECM (3D PCL scaffold with fibrillar FN structure) achieved a superior cell proliferation rate, compared to standard 3D models like hydrogels and conventional 3D-printed scaffolds^24,25^. The reason lies in the 3D EECM’s unique fibrillar design, which provides a larger surface area for cell attachment and greater porosity compared to other systems. In addition, the increased height of our PCL scaffold also contributes to this by offering additional space for cell migration, penetration, and growth, facilitated by the integration of 3D fibrillar FN. Furthermore, we utilized electro-assisted jet writing technology to precisely control the pore size of the PCL scaffold, crucial for cell motility and effective nutrient exchange throughout the scaffold. In this study, we used an optimized PCL scaffold which has 500 μm of pore size and 20 stacks (200 μm) of height. Interestingly, given their epithelial morphology, the CCL-247 cells typically exhibit greater sensitivity to attachment conditions and lose their attachability more easily than the fibroblast-like CT26 cells, as seen in their reduced viability on the TCPS beyond day 4. This observation can be attributed to our reassessment, which indicated that overconfluent conditions—leading to contact inhibition and nutrient depletion—are likely exacerbating factors. However, these same CCL-247 cells demonstrated robust proliferation on the 3D EECM. This outcome implies that the EECM provides not just a relief from the spatial and nutritional constraints inherent to 2D systems like TCPS but also offers an environment that supports strong cell adhesion, thereby mitigating the cells’ heightened vulnerability to detaching and to potential toxicities. On Day 8, CCL-247 demonstrated significant expansion to 2.64 million cells on 3D EECM which is comparable with the CT-26 mouse cancer cell on the 3D EECM (Fig. 1A).

### 3D EECM expanded cells demonstrate successful electroporation

**Figure 2:**
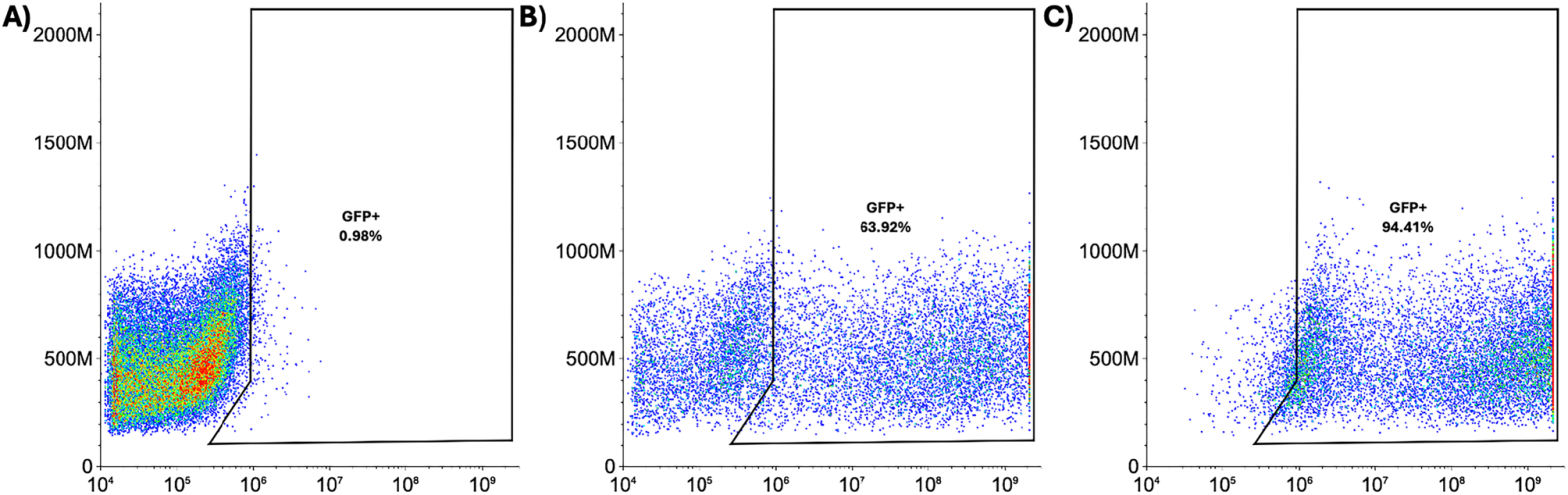
Transfection Efficiency of CCL-247 cells. Cells were transfected with GFP plasmid. The percentages of GFP-expressing cells were determined by flow cytometry. (**A**) Electroporated cells cultured on 3D EECM without GFP plasmid, (**B**) Electroporated cells cultured on 3D EECM with GFP plasmid, (**C**) Electroporated cells cultured on TCPS with GFP plasmid.

Following the expansion of CCL-247 on 3D scaffolds, the ability of the cells to efficiently express the transfected plasmid was assessed. To investigate this, a GFP plasmid was utilized in order to sort cells using flow cytometry. Cells were grown on the 3D EECM and the TCPS for 8 days to a concentration of 2.64 × 10^6^ cells per single scaffold. Cells were dissociated from the matrix by enzymatic and manual dissociation using collagenase-I and proper pipetting. Following dissociation, cells were electroporated using the same process that is used for Vigil manufacturing with 1 × 10^7^ live cells per cuvette. Forty-eight hours following electroporation, cells were sorted by flow cytometry using a ZE5 Cell Analyzer (Bio-Rad, USA) with an EGFP signal. Cells grown on 3D EECM scaffolds retained the ability to be electroporated and express GFP. Transfection efficiency was comparable between 3D EECM and TCPS-derived cells (63.92% vs. 94.41%) (Fig. 2).

### Large-scale CCL-247 cell expansion using 3D EECM

**Figure 3:**
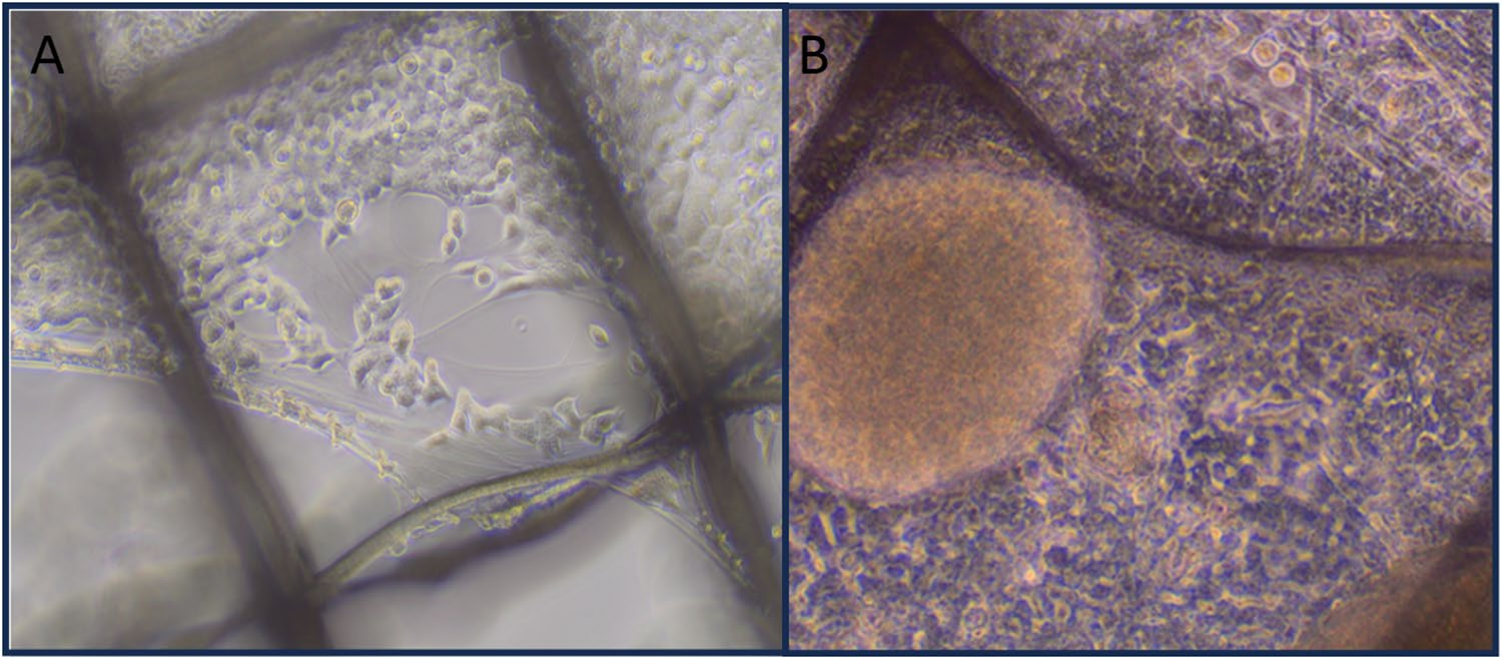
(**A**) Cell growth on scaffold matrix under 10× phase contrast microscopy at Day 2 and (**B**) Day 6.

Following successful small-scale expansion and transfection, CCL-247 cancer cells were seeded onto the 3D EECM for large-scale Vigil manufacturing. Scaffolds (n = 60) were placed individually in each well of a 12-well plate and seeded at 2.42 × 10^5^ per scaffold (14.5 × 10^6^ CCL-247 cells in total). Cells were visualized under a microscope the next day. CCL-247 cells had attached to the scaffold and began to proliferate (Fig. 3A,B). After 8 days in culture, CCL-247 cells became confluent on the scaffold. The scaffolds were transferred to a 15 mL tube with 0.5% collagenase to detach cells. After enzymatic digestion and manual dissociation, 1.21 × 10^8^ CCL-247 cells were collected which is 8.34 times increased from the initial seeded cell number within 8 days of proliferation time.

### Successful manufacturing of Vigil using 3D EECM scaffold cells

To demonstrate that cancer cells amplified using 3D engineered scaffold can be used for large-scale Vigil production, we performed side by side comparison using cancer cells amplified with 3D scaffold and standard TCPS culture using tissue culture flasks. CCL-247 cells have been well characterized in the Vigil manufacturing process, and it was used as model cell line in establishment Vigil manufacturing SOPs and method validation.

**Figure 4:**
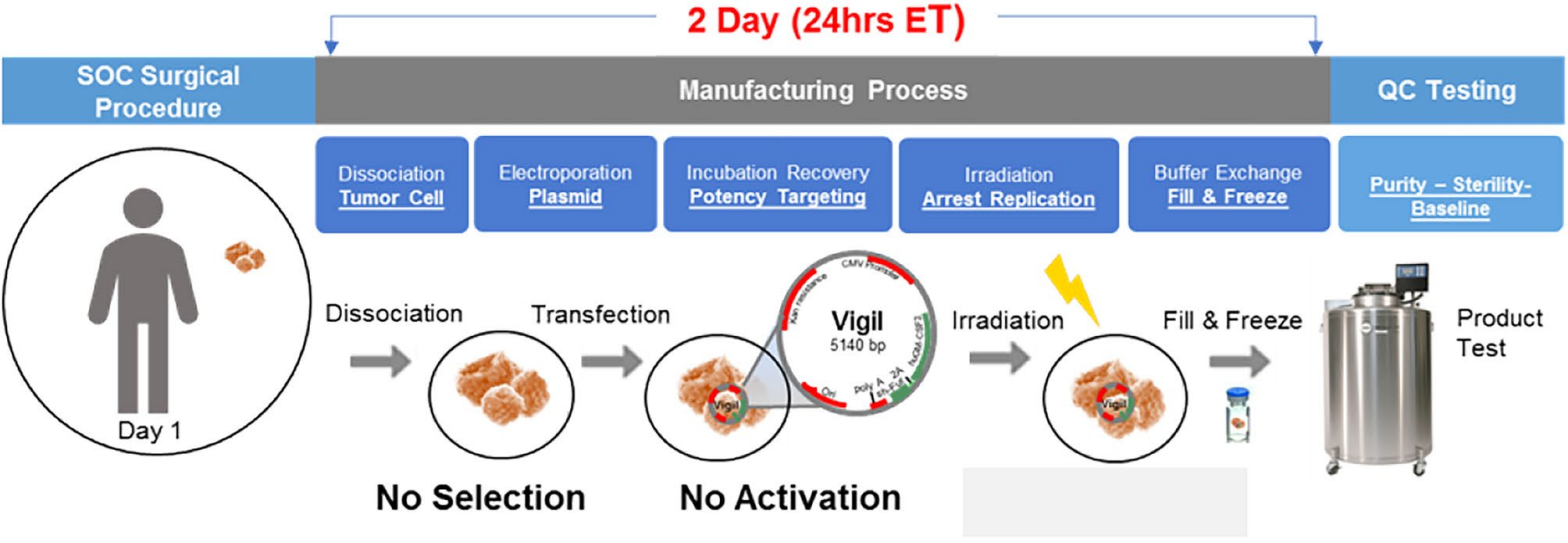
Vigil manufacturing process.

Following harvest, cells were subjected to the Vigil manufacturing process (Fig. 4).

### Vigil produced from cells expanded on 3D scaffold passes cytokine product release criteria

A critical feature of Vigil is the increased production of GM-CSF and decreased production (knockdown) of TGF-β1 when compared to the starting cells. GM-CSF production level of ≥ 30 pg/million cells and > 30% knockdown of TGF-β1 are two key criteria for Vigil manufacturing release necessary for clinical product to be administered to patient candidate as determined with FDA guidance^9^. To investigate the GM-CSF and TGF-β1 level in both 2D and 3D CCL-247 cell derived Vigil product, 1.33 × 10^6^ cells were sampled from both expansion types to create QC samples at pre-electroporation (Pre-TXF), and post-irradiation (Post-IRR) process timepoints. These QC samples were processed in identical conditions which are used for Vigil product testing. Cells are cultured in 1 mL X-VIVO 10 media for 4 days, then media samples (supernatant) were collected for cytokine analysis using ELLA, an automated ELISA platform.

**Figure 5:**
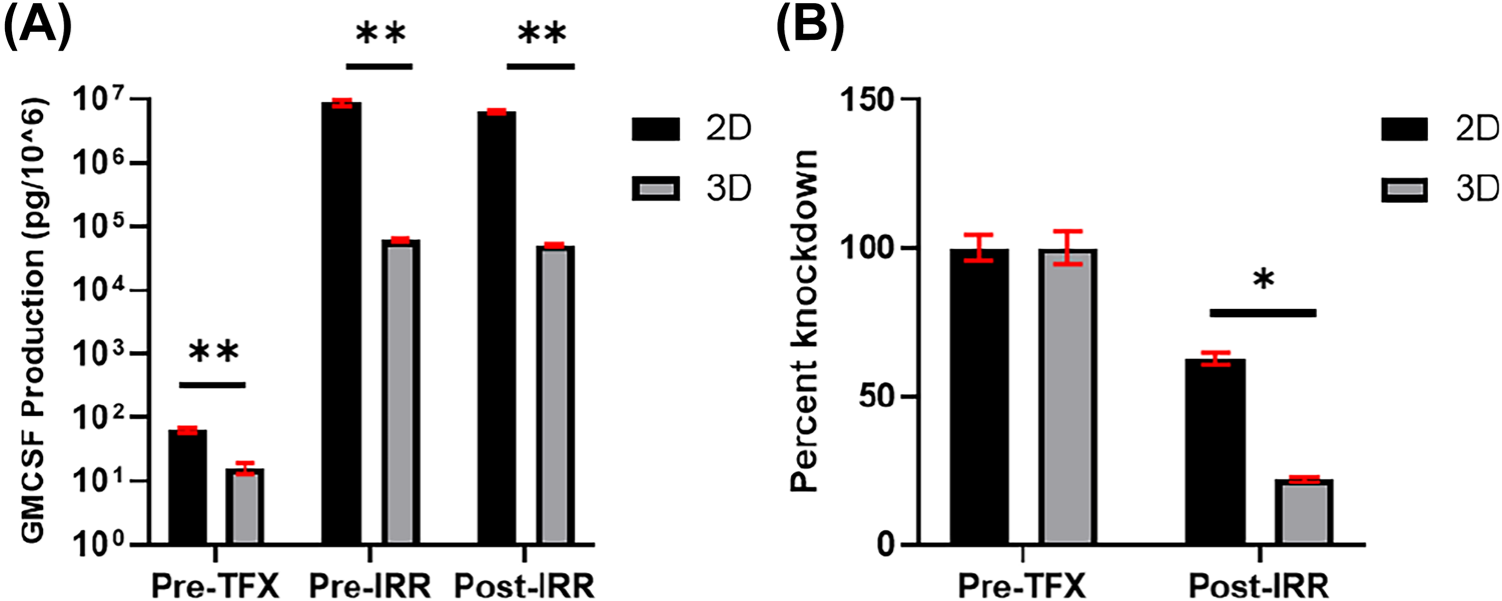
GM-CSF (**A**) and TGF-β1 (**B**) Cytokine Expression level pre-treatment (Pre TFX) versus post irradiation (Post IRR). *p = 0.0001, **p < 0.000001.

The production level of GM-CSF for CCL-247 cells prior to Vigil plasmid electroporation (Pre-TFX sample) was 63.6 ± 5.5 pg/10^6^ and 16.3 ± 3.5 pg/10^6^ respectively for 2D and 3D cells. Following transfection, GM-CSF production increased more than 3000 fold from 63.6 to 6,317,610.3 pg/10^6^ and 16.3 to 51,433.1 pg/10^6^ after irradiation (Post-IRR sample) (Fig. 5A). Although the expression of GM-CSF in transfected 3D EECM-derived cells was 100-fold lower than GM-CSF level of 2D-derived cells, the GM-CSF level in 3D EECM-derived cells surpassed GM-CSF release criteria, which is 30 pg/mL for post-irradiation samples. The marked increase of GM-CSF expression in both 2D and 3D cells suggested that the transfection and expression of Vigil plasmid was successful in both CCL-247 cells from 2D tissue culture flask and 3D scaffold.

To investigate the knockdown of TGF-β1, the active form of TGF-β1 was released from X-VIVO 10 media samples using acid treatment and was detected by antibodies specific to active TGF-β1 using automated ELISA platform: ELLA. Both 2D and 3D CCL-247 cells produce comparable amounts of active TGF-β1 prior to transfection at 13,144.36 ± 925.11 pg/10^6^ and 13,321.30 ± 1165.65 pg/10^6^ respectively (data not shown). The active TGF-β1 level remained at 8272.81 ± 96.35 pg/10^6^ for 2D cells after irradiation (Post-IRR samples) and further decreased to 2931.83 ± 75.83 pg/10^6^ for 3D cells after irradiation (Post-IRR samples) (data not shown). The percentage of TGF-β1 knockdown was 37% for 2D cells at Post-IRR. The percentage of TGF-β1 knockdown for post-IRR samples of 3D scaffold cells 78% respectively (Fig. 5B). Indicating both conditions met the 30% knockdown Vigil product release criteria.

#### A comprehensive set of Vigil manufacturing release criteria was met using 3D EECM derived cells

CCL-247 cells from 3D EECM yielded similar results to CCL-247 cells from 2D culture during the Vigil manufacturing process. Viability before electroporation, viability after electroporation, and the overall cell recovery rate after electroporation, were similar between the two groups and passed product release criteria (Table 1).

Importantly, all product release criteria for Vigil manufacturing were achieved using CCL-247 cells from 3D scaffold (Table 2). The overnight percentage recovery of 3D scaffold was 55%, which was similar to 2D results, suggesting that successful Vigil manufacturing using 3D EECM is of high likelihood with subsequent study. Further, sterility analysis of the vialed product also proved that final product manufactured using 3D scaffold cells was sterile. The stability analysis results show that final products from 3D scaffold were similar to products from 2D culture cells. All these results prove that 3D scaffold culturing method could be used to amplify cancer cells to support large-scale Vigil production.

**Table 2:** Vigil manufacturing specifications and results.

Release criterion	Release test	Sampled from	Specifications	2D pass/fail	3D pass/fail
Identity	Vigil plasmid verification	Final product	Detected	Pass	Pass
Potency	GM-CSF protein production	In-process	≥ 30 pg/million cells increase	Pass	Pass
	TGF-β1 protein production	In-process	≥ 30% knockdown or undetectable	Pass	Pass
Potency/quantity	Intact cell concentration	In-process	1.0 × 10^6^ cells/mL 1.0 × 10^7^ cells/mL	Pass	Pass
Viability	Intact cells	In-process	≥ 70% intact cells	Pass	Pass
Purity/safety	USP <85> Endotoxin	Final product	≤ 5 EU/mL	Pass	Pass
	21 CFR 610.12 Sterility	Final Product	Negative	Pass	Pass
	USP <63> Comparable Mycoplasma	In-process	Not detected	Pass	Pass

## Discussion

We assessed clinical release criteria of the Vigil manufacturing process approved by FDA and utilized for clinical trial participation^5,8,9^. Furthermore, a side-by-side comparison of Vigil immunotherapy manufacturing was also made using CCL-247 cancer cells expanded from traditional 2D cell culture on TCPS to the 3D EECM scaffold approach. Our results demonstrated cells derived from 3D scaffold were suitable for Vigil immunotherapy manufacturing, and both the 3D EECM scaffold and standard 2D culture products met FDA-approved QC release criteria. The 3D scaffold culture conditions did not appear to alter cell characteristics during the manufacturing process^16^. This was demonstrated through comparison of cell viability, recovery rate after electroporation, and transfection efficiency. Most importantly, the increased GM-CSF cytokine expression level and knockdown of TGF-β1 expression from Vigil product manufacture were well within release thresholds, meeting QC release criteria. Additionally, the final products manufactured using 3D scaffold passed all safety testing. This demonstrates that Vigil manufactured with cells expanded from 3D EECM scaffold can meet QC sterility criteria and that the expanded 3D scaffold material culturing process can be performed under our current conditions.

Although preliminary work suggests that the scaffold derived cells do not disturb the manufacturing process to construct Vigil, multiple scaffolds were seeded and subcultured only once over a period of 1-week prior to start of the manufacture. This work was conducted using an established surrogate cell line. Tests will need to be developed to assure that cells grown on the scaffold are not only suitable for manufacturing process but possess a similar genetic finger-print to the original cells dissociated from the tumor biopsy. Scaffold ligand and growth media development will also be critical components to achieve this end.

Currently, Vigil immunotherapy manufacturing does not contain a cell expansion step. Therefore, a minimum of 10–30 g (golf ball size) amount of tissue is required. During the Phase IIb trial of Vigil versus placebo in newly diagnosed Stage III/IV ovarian cancer patients, manufacturing failure occurred for some patients due to insufficient cell number. The utilization of 3D scaffold expansion would increase the number of cells for Vigil manufacture thus reducing manufacturing rates due to insufficient cells. Another important characteristic of Vigil is the display of clonal neoantigens. Increasing the amount of cells available for Vigil manufacture would therefore potentially increase the amount of clonal neoantigens present for dendritic cell uptake, processing and presentation on the cell surface for recognition by effector T cells^26^.

Synthetic and/or allogeneic neoantigen based cancer immunotherapy approaches have mixed results in the clinic, possibly related to use of non-immunogenic and/or subclonal neoantigens. However, with autologous cancer tissue based immunotherapies the immunogenic neoantigens are potentially more likely preserved, but the ability to obtain sufficient tissue on all patients is difficult. Identification of neoantigens for synthetic immunotherapy through bioinformatics and construction of mRNA based therapeutics risks lack of personal specificity and relevance to the patient^26^. In a recently completed Phase 1/2 trial in advanced/metastatic solid tumors, a self-amplifying mRNA targeting 20 common driver mutations delivered with a chimp adenovirus combined with ipilimumab and nivolumab demonstrated T cell activation toward TP53 mutations^27^. However, the response rate observed was 0%. Another all neoantigen based therapy, mRNA-4157 (V940) uses a bioinformatics pipeline to identify up to 34 unique neoantigens. In a Phase 2b study of mRNA-4157 (V940) combined with pembrolizumab in completely resected high-risk melanoma patients, a tolerable safety profile was demonstrated. In addition, there was suggestion that RFS was improved in the combination therapy group compared to pembrolizumab alone (HR 0.561; two-tailed p = 0.053) but preliminary analysis has not yet demonstrated impact on overall survival^28^. While neoantigen therapies developed through identification of common driver mutations, or through bioinformatics pipelines represent significant advances in the field, they may be missing optimal relevant and essential neoantigens provided through use of autologous tissue. Specifically the clonal neoantigens and the full individualized neoantigen repertoire^26^. Extensive retrospective clinical data support relationship of clonal TMB and clonal neoantigen to overall survival advantage^29,30^. Particularly in response to checkpoint inhibitor therapy^29,31–35^. Perspective demonstration of clonal neoantigen or surrogate cTMB would seem warranted. Limited cellular expansion with scaffold based manufacturing combined with clonal neoantigen or cTMB targeting is a reasonable future approach to consider in cancer patients with limited tissue for Vigil construction.

## Conclusion

Successful demonstration of limited expansion of CCL-247 cells on 3D EECM was achieved. Additionally 3D EECM derived cells retained transfection ability and passed all Vigil related manufacturing criteria. In addition, current Vigil manufacturing for ovarian cancer requires harvesting of tumor tissue through standard of care surgical procedure, however with scaffold expansion needle core biopsies which involve a less risky procedure could be utilized as a starting material to produce Vigil immunotherapy. In the Phase 2b VITAL trial, a minimum of 4 manufactured doses was required for entry into the study. Limited cell expansion would also not only further increase the number of patients who are suitable for Vigil production but would possibly expand the use of Vigil to other less bulky or unresectable diseases such as pediatric cancers, earlier stage cancer, and limited accessible cancer cells contained in ascites and/or pleural effusion following further FDA IND development. In addition, limited cell expansion could provide additional doses allowing for the study of Vigil boost treatments to potentially extend clinical benefit if achieved. Following the initial 4–12 doses of Vigil administered monthly, additional doses could be manufactured and administered on an extended schedule. By boosting the immune system to activate the memory T cell response, patients may achieve a longer progression free interval. Additionally, a broader range of patients could be constructed if less material harvested could be expanded. Vigil would then be able to be more easily advanced to earlier disease stage opportunity. It may also be possible with further study and condition modification that a richer tumor subpopulation could be expanded with higher concentration of the clonal neoantigen profile or lower diluting signal of subclonal neoantigen profile. Thereby enhancing potency of each therapeutic dose using such a source for Vigil construction^26^.

## Data Availability

The data that support the findings of this study are available from the corresponding author upon reasonable request.
